# Efficient soluble PTCBI-type non-fullerene acceptor materials for organic solar cells

**DOI:** 10.1007/s12200-023-00063-6

**Published:** 2023-04-23

**Authors:** Xiang Gao, Fengbo Sun, Xinzhu Tong, Xufan Zheng, Yinuo Wang, Cong Xiao, Pengcheng Li, Renqiang Yang, Xunchang Wang, Zhitian Liu

**Affiliations:** 1 Hubei Engineering Technology Research Center of Optoelectronic and New Energy Materials, Hubei Key Laboratory of Plasma Chemistry and Advanced Materials, School of Materials Science and Engineering, Wuhan Institute of Technology, Wuhan, 430205 China; 2 Key Laboratory of Optoelectronic Chemical Materials and Devices (Ministry of Education), School of Optoelectronic Materials & Technology, Jianghan University, Wuhan, 430056 China; 3 Wuhan National Laboratory for Optoelectronics, Huazhong University of Science and Technology, Wuhan, 430074 China

**Keywords:** Non-fullerene acceptor, Soluble PTCBI, Organic solar cells

## Abstract

Single perylene diimide (PDI) used as a non-fullerene acceptor (NFA) in organic solar cells (OSCs) is enticing because of its low cost and excellent stability. To improve the photovoltaic performance, it is vital to narrow the bandgap and regulate the stacking behavior. To address this challenge, we synthesize soluble perylenetetracarboxylic bisbenzimidazole (PTCBI) molecules with a bulky side chain at the bay region, by replacing the widely used “swallow tail” type alkyl chains at the imide position of PDI molecules with a planar benzimidazole structure. Compared with PDI molecules, PTCBI molecules exhibit red-shifted UV–vis absorption spectra with larger extinction coefficient, and one magnitude higher electron mobility. Finally, OSCs based on one soluble PTCBI-type NFA, namely MAS-7, exhibit a champion power conversion efficiency (PCE) of 4.34%, which is significantly higher than that of the corresponding PDI-based OSCs and is the highest PCE of PTCBI-based OSCs reported. These results highlight the potential of soluble PTCBI derivatives as NFAs in OSCs.

**Graphical abstract:**

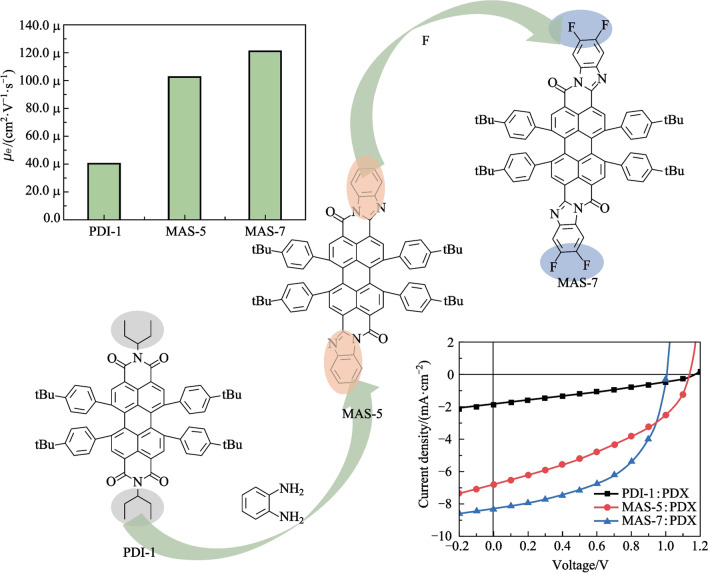

**Supplementary Information:**

The online version contains supplementary material available at 10.1007/s12200-023-00063-6.

## Introduction

The emergence of new and efficient non-fullerene acceptors (NFAs) has driven the development of organic solar cells [1–11]. NFAs have been widely investigated due to strong absorption in the UV–visible-near infrared (NIR) range and tunable energy levels [12–16]. Among the different types of NFAs, perylene diimide (PDI)-based NFAs have the advantages of low synthesis cost and good stability [17–20]. In the last decade, PDI acceptors have evolved considerably [21–23], mainly by linking multiple PDI units to a crowded core, thus distorting the molecules and disrupting the strong self-aggregation tendency [24–29]. Recently, the power conversion efficiency (PCE) based on PDI-type NFAs has exceeded 11% [30–33].

Considering its good stability and low cost, which is also important for the commercialization of OSCs, PDI is still very enticing when design novel NFAs. In particular, monomeric PDIs, which can be obtained in limited synthesis steps, and can be produced at significantly lower costs with greater stability than non-PDI type acceptors. However, the development of OSCs based on monomeric PDIs in the last few years has been limited by low short-circuit current density (*J*_sc_) and small fill factor (FF), mainly because the wide distribution of π-π* stacking configurations increases the energetic disorder trap [34–37]. Therefore, it is very important to regulate the stacking of monomeric PDI molecules. However, only the steric hindrance at the bay positions has been paid great attention in previous studies: the steric hindrance of “swallow tail” type alkyl side chains at the imide position has been overlooked although the branching positions of alkyl chains have great influence on the optoeletronic properties of conjugated materials [34, 38–43].

Among the different kinds of PDI derivatives, fully fused perylenetetracarboxylic bisbenzimidazole (PTCBI) possesses planar end groups, and it is also the first n-type molecule to construct heterojunction OSCs, which has opened up an era of OSC research and applications [44]. Because there are no alkyl groups in PTCBI, it is possible to construct planar heterojunction solar cells only by evaporation. Therefore, the PCE of OSCs using PTCBI as acceptors lagged behind that of other NFAs-based OSCs [45–49] because PTCBI could not be used in high-efficiency bulk heterojunction OSCs due to its insolubility.

**Fig. 1:**
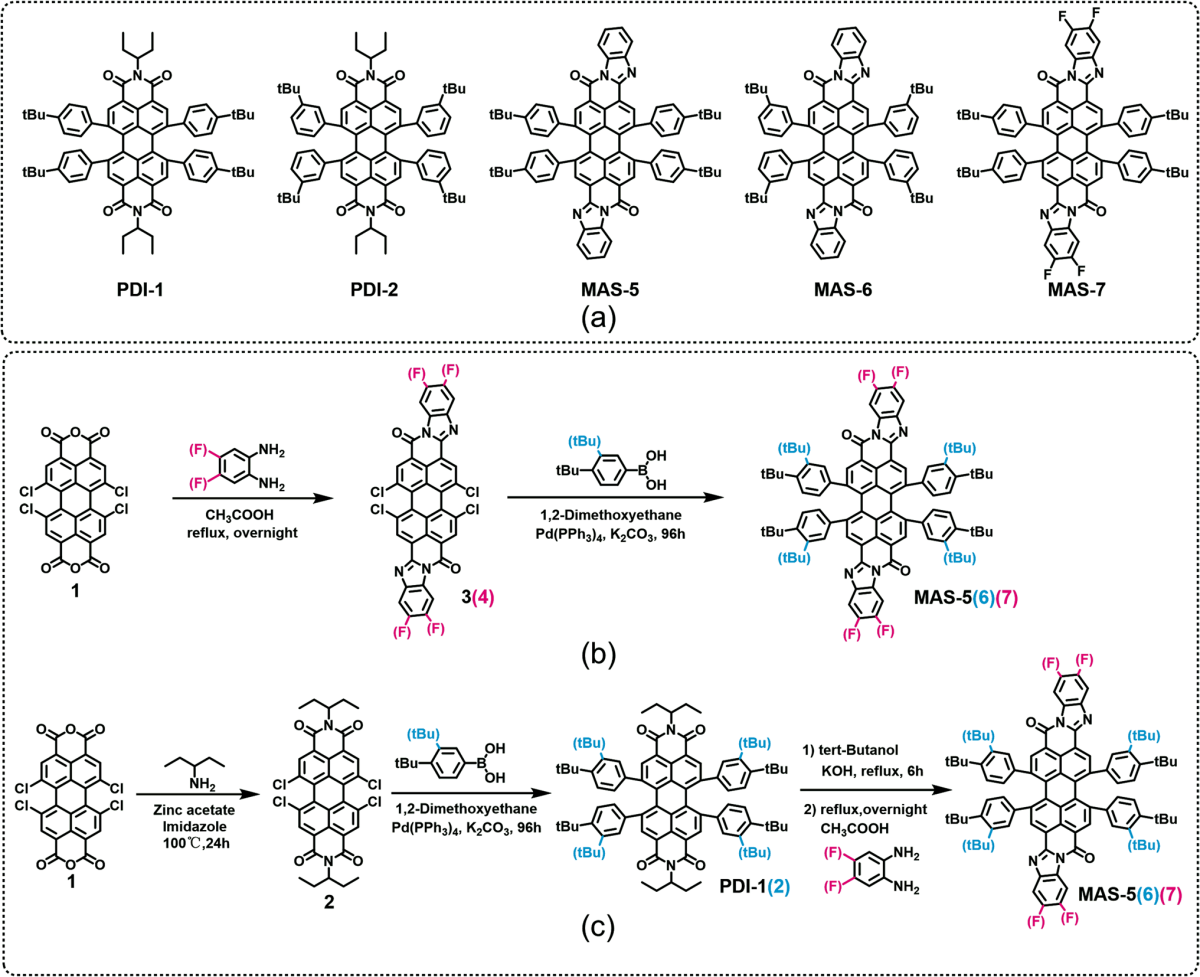
**a** Molecular structures of PDI-1, PDI-2, MAS-5, MAS-6 and MAS-7. The synthetic route I (**b**) and II (**c**) of MAS-5, MAS-6 and MAS-7

In this work, the insolubility of PTCBI-type molecules was solved by attaching four tert-butylphenyl groups to the molecular bay position. Moreover, the large steric hindrance introduced by the tert-butylphenyl groups in the middle part of PTCBI might modulate the PTCBI molecule packing as A-D-A-type NFAs [50, 51]. The optical properties and photovoltaic performance of three PTCBI-type materials and two small-molecule PDI-type materials (as shown in Fig. 1a) were compared when PTB7-Th and chlorinated PTB7-Th(PDX), were selected as the donor materials (Fig. S1). Compared with pure film of one PDI-type acceptor (PDI-1), the electron mobility of PTCBI-type NFA in pure films were founded to be one order of magnitude higher. Devices based on MAS-7 (PTCBI-type NFA), exhibited a PCE of 4.34% with an open-circuit voltage (*V*_oc_) of 1.00 eV, a short-circuit current density (*J*_sc_) of 8.26 mA/cm^2^, and a fill factor (FF) of 52.41%. This PCE value was the highest among PTCBI-based OSCs, and was also higher than corresponding devices based on PDI-type NFAs. These results highlight the potential of soluble PTCBI derivatives as the NFAs in OSCs.

## Results and discussion

The molecular structures of two PDI-type small-molecule materials (PDI-1, PDI-2) and three soluble PTCBI-type small-molecule materials (MAS-5, MAS-6, MAS-7) are shown in Fig. 1a. Four tert-butylphenyl groups were attached at the PDI bay position in order to ensure good solubility. The positions of tert-butyl groups in phenyl were varied to fine-tune the steric hindrance effects. MAS-5, MAS-6 and MAS-7 could be obtained by a two-step synthesis as shown in Fig. 1b. First the 4Cl-PTCBI was obtained by the condensation reaction of 1,6,7,12-tetrachloroperylene tetracarboxylic acid dianhydride with o-phenylenediamine, and then the 4-tert-butylphenyl groups were attached via Suzuki coupling reaction. Because 4Cl-PTCBI (compounds 3 and 4 in Fig. 1b) is insoluble and difficult to purify, the total yields of MAS-5, MAS-6 and MAS-7 were as low as 5%. Therefore, the synthetic route for MAS-5 and MAS-7 was optimized as shown in Fig. 1c with PDI-1 or PDI-2 as the intermediate product. The alkyl chain of PDI-1 was removed in the presence of a strong base and the product, perylene dianhydride, was used directly and condensed with o-phenylenediamine. Although the synthesis route was longer, the yield of each reaction was higher, and the total yield was increased to about 45%. The final products were verified to be structurally correct by ^1^H-NMR and MS. Detail data could be found in the supporting information. Note that PDI-1, PDI-2, MAS-5, MAS-6, and MAS-7 were found to be soluble in chloroform (> 10 mg/mL) and chlorobenzene (> 12 mg/mL) at room temperature.

**Fig. 2:**
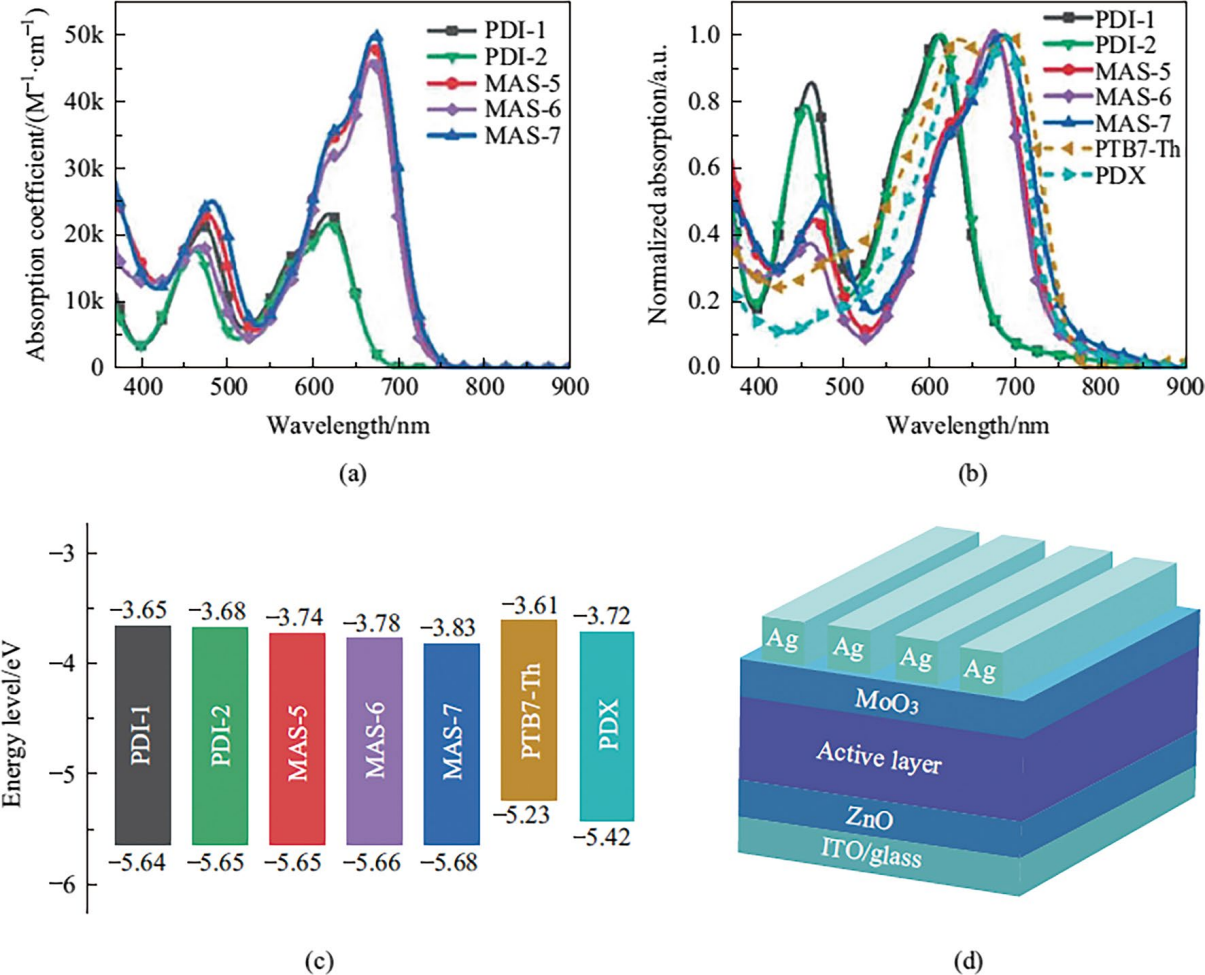
**a** UV–vis absorption coefficients of PDI-1 (black line), PDI-2 (green line), MAS-5 (red line), MAS-6 (violet line) and MAS-7 (blue line) in dilute chloroform solution. **b** Normalized UV–vis absorption spectra of PDI-1, PDI-2, MAS-5, MAS-6, MAS-7 and PTB7-Th (yellow dashed line)/PDX (cyan dashed line) in films. **c** Schematic diagram of the energy levels of the donor and acceptor materials. **d** The inverted device structure used in this work.

Figure 2a and b show the absorption spectra of PDI-1, PDI-2, MAS-5, MAS-6 and MAS-7 in dilute chloroform solution and in films, respectively. Relevant parameters are summarized in Table 1. All PTCBI-type and PDI-type small-molecule materials showed bimodal absorption peaks. The peak in the 450–500 nm range originated from the π-π* transition of the twisted backbone [21–23, 52–56]. Compared with PDI-1 (475 nm), the peaks of MAS-5 and MAS-7 were 479 and 484 nm, respectively. This red shift may be attributed to the increase in the backbone length. Comparing PDI-1 with PDI-2 and MAS-5 with MAS-6, the π-π* transition peaks of molecules with 3-tert-butylphenyl groups (PDI-2 and MAS-6) blue-shifted compared with those with 4-tert-butylphenyl groups (PDI-1 and MAS-5). This phenomenon could be attributed to the larger steric hindrance of 3-tert-butylphenyl groups. This result was consistent with the dihedral angle of density function theory (DFT) calculation results, as discussed below. Meanwhile, the peaks in the longer wavelength region could be attributed to the intramolecular charge transfer (ICT) effect which was evidenced by the temperature-dependent UV–vis absorption spectra and polarity-dependent fluorescent spectra of those molecules. As shown in Fig. S2a, the height ratio of the shoulder peak at 620 nm to the main peak at 670 nm does not change with temperature, suggesting that the absorption spectra indicate the intrinsic molecular property rather than the aggregations. In addition, the emission spectra of each small molecule gradually became red-shifted and broadened with the enhancement of solvent polarity, as shown in Fig. S2b–f, which is attributed to the obvious ICT effect [57]. The ICT absorption peaks of PTCBI-type molecules MAS-5, MAS-6 and MAS-7 red-shifted about 60 nm relative to that of PDI-1 and PDI-2. This might be because the lowest unoccupied molecular orbital (LUMO) of PTCBI-type molecules is deeper [45]. The molar extinction coefficients of PTCBI-type NFAs at the maximum absorption peak were twice more than those of PDI-type NFAs as listed in Table 1. Unlike the case for steric hindrance effects on the π-π* transition, varying the position of *t*-butyl groups only slightly decreased the molar extinction coefficients, but did not shift the ICT peaks. The fluorinated molecule MAS-7 had only a 3 nm red-shift in solution compared with the non-fluorinated one MAS-5, although the fluorine atoms belong to electron-withdrawing groups. This was because the fluorine atoms barely contributed to the LUMO, as demonstrated by the DFT calculations.

**Table 1:** Summary of photophysical and electrochemical properties of the NFAs

Compound	*λ*^a^ /nm	*λ*_max_/nm		*ε*_max_^b^ /(M^−1^·cm^−1^)	*E*_LUMO_^c^ /eV	*E*_HOMO_^c^ /eV	*E*_g_^c^ /eV
		Solution	Film				
PDI-1	475	619	609	2.3 × 10^4^	− 3.65	− 5.64	1.99
PDI-2	464	620	614	2.2 × 10^4^	− 3.68	− 5.65	1.97
MAS-5	479	670	673	4.8 × 10^4^	− 3.74	− 5.65	1.91
MAS-6	470	670	673	4.6 × 10^4^	− 3.78	− 5.66	1.88
MAS-7	484	673	683	5.0 × 10^4^	− 3.83	− 5.68	1.85

^a^π-π* transition of the deformed backbone in solution, ^b^Measured in chloroform solution with a concentration of 10^−5^ M, 1 M = 1 mol/L, ^c^Determined by CV

The electrochemical properties of these molecules were investigated by cyclic voltammetry (CV). Corresponding CV curves were showed in Fig. S3a. The LUMO and highest occupied molecular orbital (HOMO) energy levels of PDI-1, PDI-2, MAS-5, MAS-6 and MAS-7 were calculated using the reduction/oxidation starting points of their CV curves, which were − 3.65/ − 5.64, − 3.68/ − 5.65, − 3.74/ − 5.65, − 3.78/ − 5.66, and − 3.83/ − 5.68 eV, respectively (Table 1). Different tert-butyl sites had negligible effects on the molecular energy levels. The PTCBI-type molecules exhibited deeper LUMO energy level than the corresponding PDI-type ones, resulting in a reduction of the molecular bandgap. Fluorination did decrease the energy levels of MAS-7 because of its strong electron-withdrawing ability. However, introducing fluorine atoms did not decrease the LUMO energy levels as much as it does in other fluorinated materials [15, 58]. This was because of the limited electron distribution of LUMO on the fluorine atoms as exhibited in the results of DFT calculations. The HOMO energy levels of the polymeric donor materials PTB7-Th and PDX (Fig. 2c) were also tested by CV (Fig. S3b) and the LUMO energy levels were determined by the sum of LUMO energy level and the optical bandgap. The energy levels were − 3.61/ − 5.23 and − 3.72/ − 5.42 eV, respectively (Table S1).

**Fig. 3:**
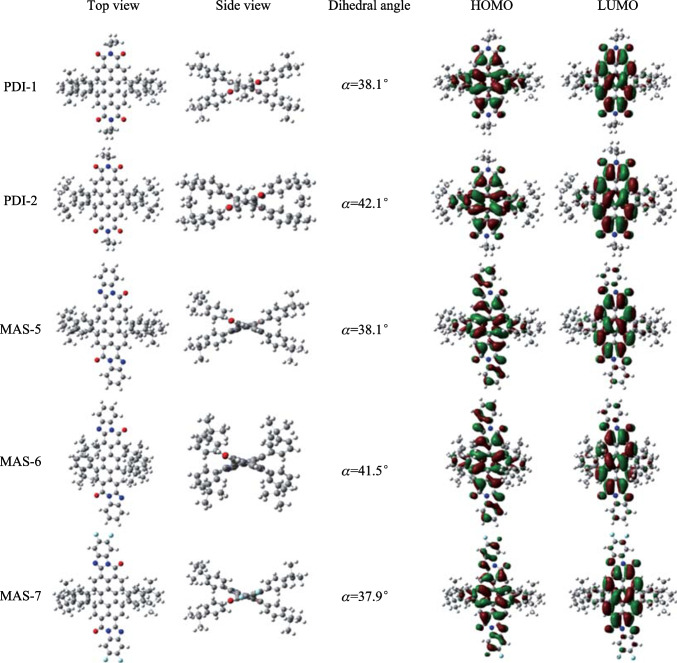
Optimized molecular conformation and frontier orbital electron distribution of NFAs based on DFT simulations

The molecular geometry and molecular frontier orbitals of the NFAs were simulated using DFT calculations at the B3LYP 6-31G level. The alkyl chain was simplified to methyl. The twist angle of the main backbone of the PDI molecule is labeled as α. The results were exhibited in Fig. 3. Obviously, the α value of molecules with 3-tert-butylphenyl groups (PDI-2 and MAS-6) was larger than that of molecules with 4-tert-butylphenyl groups (PDI-1 and MAS-5), indicating larger steric hindrance of 3-tert-butylphenyl groups. The electron distribution of the HOMO and LUMO of PDI-type molecules was mainly localized on the backbones. In contrast, the electron clouds of the HOMO levels of PTCBI-type molecules extended to the benzimidazole subunit. This fully explains the insignificant effects of fluorination on the LUMO level and the ICT absorption peaks of MAS-7. The calculated HOMO/LUMO energy levels for PDI-1, PDI-2, MAS-5, MAS-6 and MAS-7 are exhibited in Table S2, which are consistent with the CV results.

**Fig. 4:**
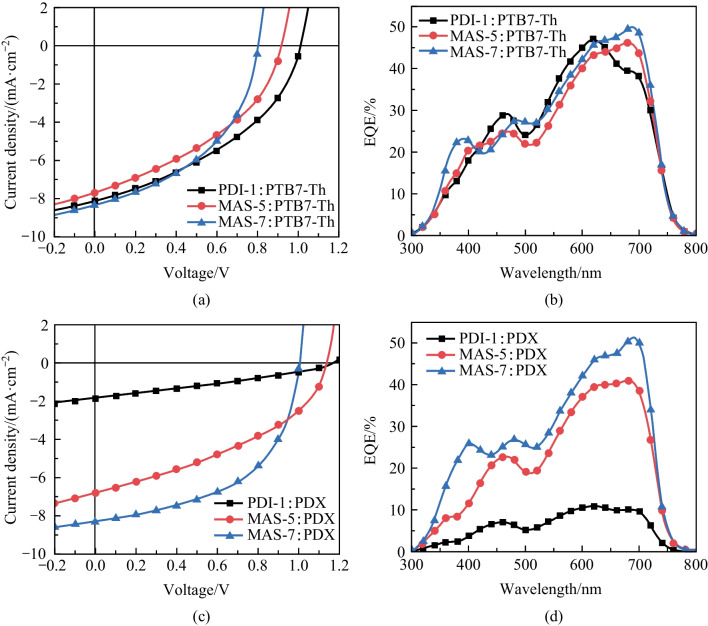
**a** *J–V* curves and **b** EQE spectra of the OSCs based on PTB7-Th:Acceptor; **c** *J–V* curves and **d** EQE spectra of the OSCs based on PDX:Acceptor

To investigate the photovoltaic performance of PDI-1, PDI-2, MAS-5, MAS-6 and MAS-7, OSCs with the inverted structure of indium tin oxide (ITO)/ZnO/active layer/MoO_3_/Ag (Fig. 2d) were fabricated. The conjugated polymers PTB7-Th and its chlorinated derivative PDX (Fig. S1) which exhibited matched energy levels as shown in Fig. 2c were selected as the donor polymers. The active layer was prepared by spin-coating the mixture of donor and acceptor with a weight ratio of 1:1 in chlorobenzene solvent containing 0.5 v% chloronaphthalene (CN) with a total concentration of 13 mg/mL. Details of device fabrication and measurements are described in the supporting information. The current density–voltage (*J–V*) curves of optimal OSCs under AM 1.5G illumination are shown in Fig. 4a and c, and the corresponding parameters are listed in Table 2. The PCEs of NFAs with 3-tert-butylphenyl groups (PDI-2 and MAS-6) were slightly lower than that of NFAs with 4-tert-butylphenyl groups (PDI-1 and MAS-5) when PTB7-Th was used as the donor material (Fig. S4a).

**Table 2:** Key parameters of OSCs based on PTB7-Th/PDX: Acceptor

Donor	Acceptor	*J*_sc_ /(mA·cm^−2^)	*V*_oc_ /V	FF	PCE	*J*_cal_ /(mA·cm^−2^)
PTB7-Th	PDI-1	8.09	1.01	40.92	3.35	7.88
	MAS-5	7.67	0.92	39.75	2.80	7.59
	MAS-7	8.32	0.81	45.08	3.02	8.25
PDX	PDI-1	1.87	1.19	30.09	0.67	1.80
	MAS-5	6.78	1.14	39.41	3.05	6.62
	MAS-7	8.26	1.00	52.41	4.34	8.12

When PTB7-Th was used as the donor material, OSCs based on PDI-1 exhibited a PCE of 3.36%, which was higher than that for OSCs based on MAS-5 (2.78%) and MAS-7 (3.02%) due to the much higher *V*_oc_. Because the *V*_oc_ could be easily improved by modifying the HOMO energy level of the donor materials [15, 58–61], the chlorinated PTB7-Th with deeper HOMO level, namely PDX, was used to improve the photovoltaic performance of PTCBI-type NFAs. As expected, those PDX-based OSCs exhibited enhanced *V*_oc_ value, thus higher PCE. The PDX:MAS-7-based device showed the best PCE (4.34%) with a larger *J*_sc_ (8.26 mA/cm^2^) and a higher *V*_oc_ (1.00 V), as well as the best FF (52.41%). PDX:MAS-5-based devices exhibited a *J*_sc_ of 6.78 mA/cm^2^, a *V*_oc_ of 1.14 V and an FF of 39.41%, with a device efficiency of 3.05%. However, the PCE of PDX:PDI-1-based OSCs dramatically decreased to 0.67% because of the significant drop in *J*_sc_. This may be due to the mismatched LUMO energy levels between the donor and acceptor. As a result, the PCE of PDX:MAS-7 (4.34%) is significantly higher than that of PTB7-Th:PDI-1 (3.35%).

The external quantum efficiency (EQE) of the optimized devices based on PDI-1, MAS-5 and MAS-7 was measured to investigate the photovoltaic response of each kind of device (Fig. 4b and d). These devices exhibited strong photoresponse in the region of 550 to 720 nm and weaker photoresponse in the region of 350 to 500 nm. MAS-5-based OSCs had a weaker response at 620 nm (43%) where PDI-1-based OSCs exhibited the maximum EQE (47%), but a stronger response at 685 nm (46%), so the difference in the integral *J*_sc_ of these two OSCs was not significant. In contrast, devices based on the fluorinated molecule MAS-7 had a similar response at 620 nm (46%) and a stronger response at 685 nm (50%) compared to devices based on MAS-5, so the MAS-7-based OSCs exhibited the larger integral *J*_sc_. However, the photovoltaic response intensity of OSCs with PDX as the donor decreased in the sequence of MAS-7, MAS-5 and PDI-1 as the acceptor. There was little change in the photovoltaic response of the MAS-7-based devices, and the integrated current density of OSCs based on PDX:MAS-7 (8.12 mA/cm^2^) was similar to those based on PTB7-Th:MAS-7 (8.25 mA/cm^2^). However, the photovoltaic response intensity of devices based on PDX:PDI-1 significantly decreased comparing to devices based on PTB7-Th:PDI-1 because of smaller offset between the LUMO energy levels, thus resulting in reduced PCE. The integral *J*_sc_ values for each device are given in Table 2, and their *J*_sc_ error values with respect to the *J*–*V* curve are within 3%.

**Fig. 5:**
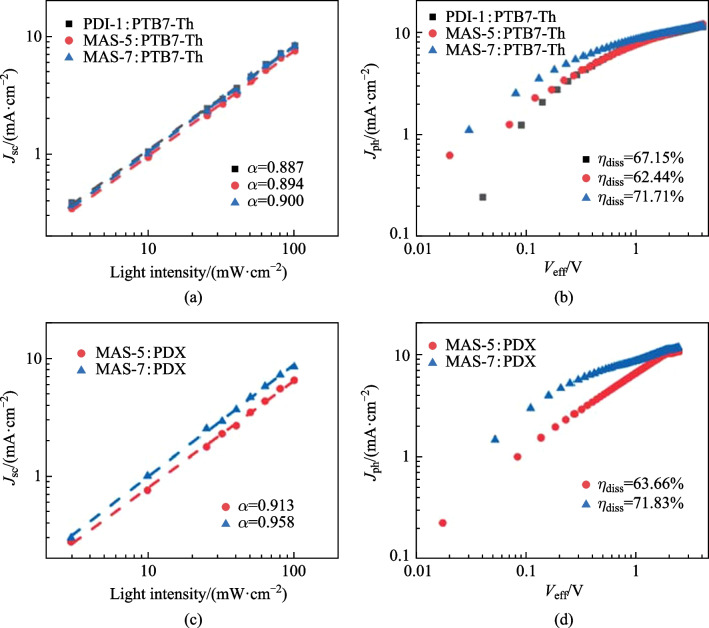
Dependence of *J*_sc_ on incident light intensity for the **a** PTB7-Th-based OSCs and **c** PDX-based OSCs and the saturated photocurrent versus effective voltage (*J*_ph_−*V*_eff_) curves of the **b** PTB7-Th-based OSCs and **d** PDX-based OSCs

The bimolecular recombination mechanism of PDI-1, MAS-5 and MAS-7-based OSCs were studied according to the relationship between *J*_sc_ and light intensity which can be described by the equation *J*_sc_ ∝ *P*_light_^*α*^. If the value of *α* is close to 1, the bimolecular recombination in OSC is negligible [28]. No objective results of devices based on PDX:PDI-1could be obtained because of the quite low PCE. As shown in Fig. 5a, the values of α calculated for the PTB7-Th:PDI-1-, PTB7-Th:MAS-5- and PTB7-Th:MAS-7-based devices were 0.887, 0.894, and 0.900, respectively, indicating the weakest bimolecular recombination in MAS-7-based devices. When PDX was used as the donor material, the α-values of MAS-5 and MAS-7 based OSCs were 0.913 and 0.958, respectively (Fig. 5c), indicating that the bimolecular recombination in the devices was suppressed to a greater extent. Therefore, the device efficiency of OSCs based on MAS-5 and MAS-7 was significantly improved.

To further investigate exciton dissociation, the relationship between photocurrent density and effective voltage was investigated, and the exciton dissociation probability (*η*_diss_) was calculated according to the equation *η*_diss_ = ${J}_{\mathrm{ph}}^{\#}$/*J*_sat_. The ${J}_{\mathrm{ph}}^{\#}$ is defined as *J*_L_ − *J*_D_ at short-circuit condition, where *J*_L_ and *J*_D_ are the current densities under illumination and in the dark, respectively. The *V*_eff_ is defined as *V*_0_ − *V*, where *V*_0_ is the voltage at zero photocurrent, and *V* is the applied voltage. *J*_sat_ is the saturated photocurrent and *V*_eff_ is dependent on the internal electric field in the OSCs. For the devices based on PTB7-Th:PDI-1, PTB7-Th:MAS-5 and PTB7-Th:MAS-7, the *η*_diss_ values were calculated as 67.15%, 62.44% and 71.71%, respectively (Fig. 5b). The PTB7-Th:MAS-7 based OSCs exhibited the maximum exciton dissociation probability. This result was also consistent with the *J*_sc_ value of corresponding devices. When PDX was used as the donor material, the *η*_diss_ values were calculated to be 63.66% and 71.83% for PDX:MAS-5- and PDX:MAS-7-based devices, respectively (Fig. 5d). These results confirmed that PTCBI-based devices exhibited more efficient exciton dissociation and suppressed bimolecular recombination compared with the PDI-based molecules. This facilitates the achievement of higher *J*_sc_ and FF.

**Fig. 6:**
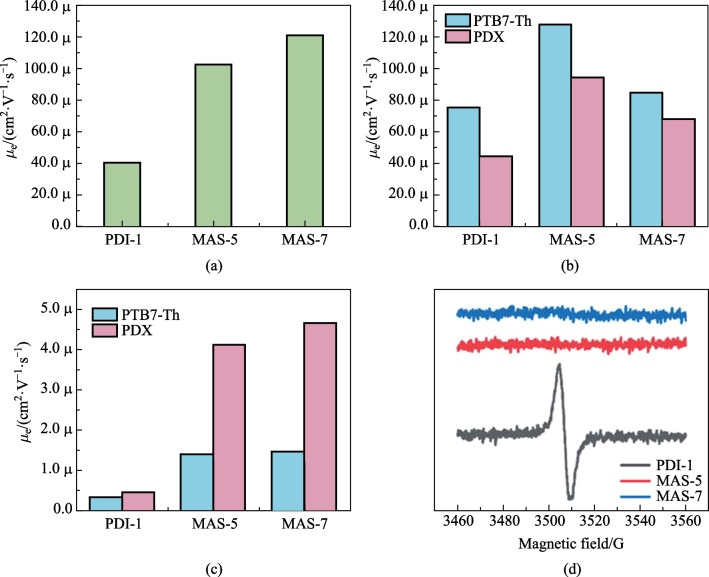
**a** Electron mobility of PDI-1, MAS-5 and MAS-7 in pure films. **b** Hole and **c** electron mobility of optimized OSCs. **d** Electron paramagnetic resonance spectra of different acceptor materials

To further investigate carrier transport characteristics, the space charge-limited current (SCLC) method was used to measure the hole mobility (*μ*_h_), using hole-only devices with the structure of ITO/PEDOT:PSS/active layer/MoO_3_/Ag, and the electron mobility (*μ*_e_) using electron-only devices with the structure of ITO/ZnO/active layer/PDINN/Ag. The mobilities were calculated according to the log *J − *log *V* curves with a slope of 2 [27], as shown in Fig. S5. In the pure molecular films, the electron mobility as shown in Fig. 6a and listed in Table S5. Obviously, electron mobility for MAS-5 and for MAS-7 was one order of magnitude higher than that for PDI-1, which might be because the bulky side groups in the middle and planar benzimidazole unit at the end of the molecules made the NFAs stack as A-D-A type molecules and then facilitated the intermolecular electron transport [50, 51]. Moreover, there was no paramagnetic behavior in the powder of MAS-5 and MAS-7. In contrast, obvious electron paramagnetic resonance (EPR) signal was observed in the powder of PDI-1. This indicated the presence of a small concentration of intrinsic radical cations or anions generated through exposure to ambient atmosphere (oxygen, water) and light in the PDI-1 powder [62], thus hindering electron transport.

The hole mobility of all devices was of the order of 10^−5^ cm^2^/(V·s) and the hole mobility of PTB7-Th-based devices was greater than that of PDX-based devices. However, the electron mobility varied around the order of 10^−7^ cm^2^/(V·s) for PDI-1-based devices and 10^−6^ cm^2^/(V·s) for MAS-5- and MAS-7-based devices. Therefore, the greater electron mobility of PTCBI-type small molecules relative to PDI facilitated the improved PCE.

**Fig. 7:**
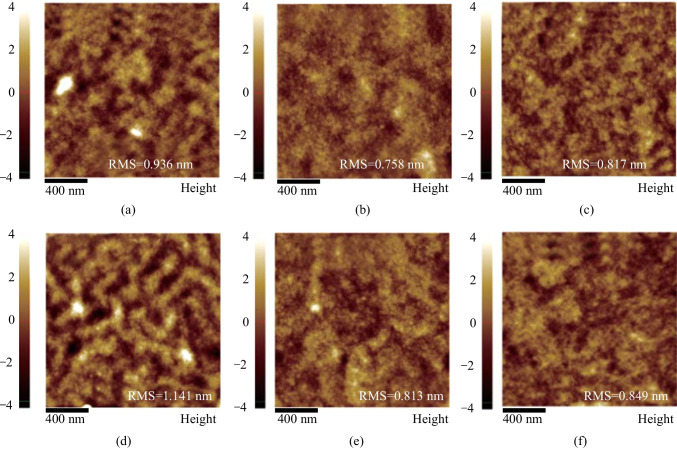
AFM images for films of **a** PDI-1:PTB7-Th, **b** MAS-5:PTB7-Th, **c** MAS-7:PTB7-Th, **d** PDI-1:PDX, **e** MAS-5:PDX, and **f** MAS-7:PDX blend

Atomic force microscopy (AFM) and transmission electron microscopy (TEM) were employed to investigate surface morphologies of the optimal blend films. As shown in Fig. 7, PDI-1/MAS-5/MAS-7:PTB7-Th blends showed smooth surfaces with root mean square (RMS) surface roughness of 0.936, 0.758, and 0.817 nm, respectively. Similarly, PDI-1/MAS-5/MAS-7:PDX blends also showed smooth surfaces, with root mean square (RMS) surface roughness of 1.141, 0.813, and 0.849 nm, respectively. The RMS of the blended film of PDX was generally larger than those of PTB7-Th, which is attributed to decreased solubility of PDX. For the acceptor material, PDI-1 exhibited the roughest surface, the PTCBI-type small molecule exhibited a smoother surface, which facilitated the contact between the interfacial layer and the active layer. Among these blend films, MAS-7-based samples showed a more appropriate roughness, which contributed to the enhancement of the FF [56]. As for the slightly larger roughness of MAS-7 relative to MAS-5, this might be attributed to decreased solubility after fluorination or stronger intermolecular interaction. The AFM images are consistent with TEM results (Fig. S6). MAS-7-based OSCs exhibited appropriate phase separation, which was conducive to higher FF and thus the improved photovoltaic performance.

## Conclusion

We synthesized three soluble PTCBI-type small-molecule materials (MAS-5, MAS-6, MAS-7) and performed a comparative study with the PDI analogs (PDI-1, PDI-2). We found that PTCBI-type materials exhibited red-shifted UV–vis absorption spectra with larger molar extinction coefficients than PDI-type materials, which is important for organic photovoltaic materials. Moreover, the electron mobility of PTCBI-type NFAs, i.e., MAS-5 and MAS-7 was one order of magnitude higher than that of PDI-1. Finally, the devices based on MAS-7:PDX exhibited the champion PCE of 4.34%, which is higher than the devices based on PDI-type NFAs. To the best of our knowledge, this is the highest PCE for PTCBI-based OSCs, presenting a breakthrough in the research of PTCBI-based OSCs. Further molecular and device engineering would undoubtedly improve the photovoltaic performance of PTCBI-type NFAs.

## Supplementary Information

Below is the link to the electronic supplementary material.

Supplementary file 1 (PDF 1298 KB)

## Data Availability

The data that support the findings of this study are available from the corresponding author, upon reasonable request.
