# Supplementary material for: Efficient soluble PTCBI-type non-fullerene acceptor materials for organic solar cells
Source: Front Optoelectron. 2023 Apr 23;16(1):8. doi: 10.1007/s12200-023-00063-6 (PMC10122612; doi:10.1007/s12200-023-00063-6)
Supplement: Supplementary file 1 — (PDF 1298 KB) [file 12200_2023_63_MOESM1_ESM.pdf]

Supplementary information

Efficient Soluble PTCBI-Type Non-Fullerene Acceptor Materials for Organic Solar Cells

Xiang Gao[1,#,*], Fengbo Sun[1,2,#], Xinzhu Tong[1], Xufan Zheng[2], Yinuo Wang[1], Cong Xiao[2], Pengcheng Li[1], Renqiang Yang[2], Xunchang Wang[2,*], Zhitian Liu[1,3,*]

[1] Hubei Engineering Technology Research Center of Optoelectronic and New Energy Materials, Hubei Key Laboratory of Plasma Chemistry and Advanced Materials, School of Materials Science and Engineering, Wuhan Institute of Technology, Wuhan 430205, China

[2] Key Laboratory of Optoelectronic Chemical Materials and Devices (Ministry of Education), School of Optoelectronic Materials & Technology, Jianghan University, Wuhan 430056, China

[3] Wuhan National Laboratory for Optoelectronics, Huazhong University of Science and Technology, Wuhan 430074, China

*Corresponding author (email: xgao@wit.edu.cn; wangxc@jhun.edu.cn; able.ztliu@wit.edu.cn)

#These authors contributed equally to this work.

Experiment Details:

Materials: All reagents and solvents, unless otherwise specified, were purchased from commercial sources and were used without further purification.

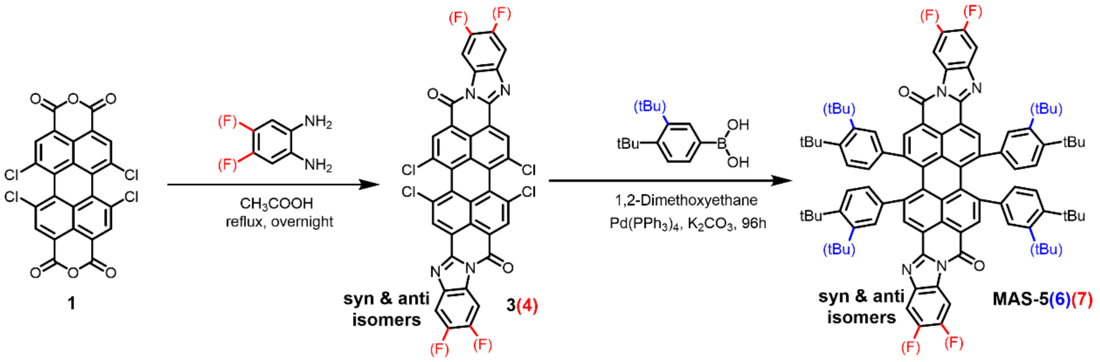

Scheme S1. Synthesis of MAZ-5, MAS-6 and MAS-7.

1) Synthesis of MAS-5

1,6,7,12-tetrachloroperylene tetracarboxylic acid dianhydride (compound 1) (0.5 g, 0.94 mmol) was suspended in 25 mL glacial acetic acid. Then o-phenylenediamine (0.22 g, 2 mmol) was added and the mixture was heated to reflux for 6 hours. After cooling to room temperature, the precipitate was filtered off and rinsed with plenty of water. It was dried in a vacuum oven at 100 °C to obtain compound 3 as an insoluble compound.

The unpurified product compound 3 (100.00 mg, 0.15 mmol), 4-tert-butylphenylboronic acid (528.05 mg, 2.97 mmol), anhydrous sodium carbonate (215.20 mg, 1.56 mmol) and tetrakis(triphenylphosphine)palladium (51.41 mg, 0.045 mmol) were added into a Schlenk tube. The nitrogen was purged three times. A mixed solution of ethylene glycol dimethyl ether (4 mL) and water (1 mL), which had been degassed, was added. The reaction was stirred at 80 °C for 48 h. After cooling to room temperature, the mixture was extracted with CH_2Cl_2, dried with anhydrous Na_2SO_4. The crude

product was purified by silica gel column chromatograph using dichloromethane/petroleum ether (2:1, v/v) as the eluent to obtain MAS-5.

2) Synthesis of MAS-6 and MAS-7

The synthesis method is the same as MAS-5

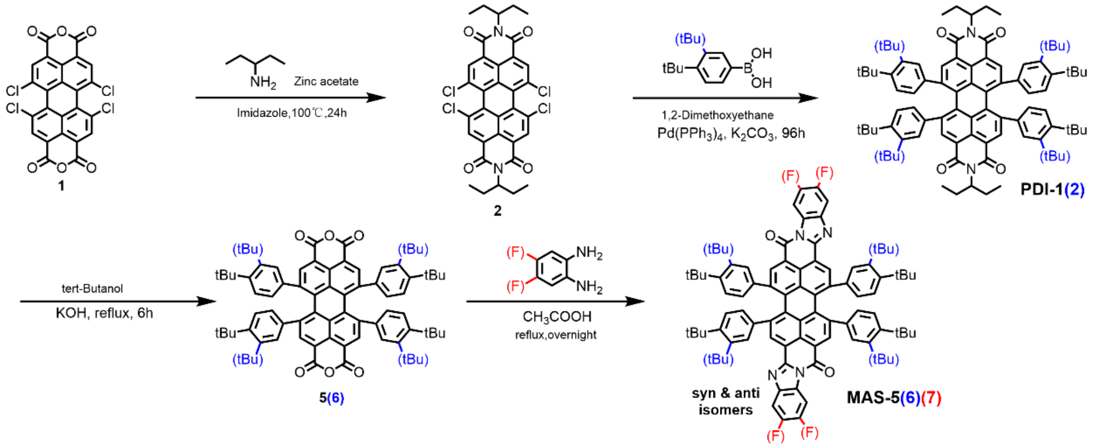

Scheme S2. Synthesis of PDI-1, PDI-2 and Optimized synthesis of MAZ-5, MAS-6 and MAS-7.

3) Synthesis of PDI-1

Compound 1 (5 g, 12.47 mmol), imidazole (40 g, 587.53 mmol) and zinc acetate (1.75 g, 9.56 mmol) were added into a 250 mL three-necked flask, and then a condenser tube and a gas guide were connected to the reaction. The temperature is gradually increased until the imidazole is melted and the rotor can be smoothly and continuously stirred. Then, 3-aminopentane (2.56 g 29.31 mmol) was added to the reaction vessel with a syringe, and the temperature was kept and stirred overnight. Then, 2 M hydrochloric acid (120 mL) was added, and the mixture was stirred for 20 min. The crude product was purified by silica gel column chromatograph using dichloromethane as the eluent to obtain Compound 2 (6.08 g, 90%)

The product compound 2 (1.00 g, 1.5 mmol), 4-tert-butylphenylboronic acid (2.13 g, 11.97 mmol), anhydrous sodium carbonate (2.17 g, 15.71 mmol) and tetrakis(triphenylphosphine) palladium (432.23 mg, 0.374 mmol) were added into a Schlenk tube. The nitrogen was purged three times. A mixed solution of ethylene glycol dimethyl ether (40 mL) and water (15 mL) which had been degassed, was added. The reaction was stirred at 80 °C for 96 h. After cooling to room temperature, the mixture was extracted with CH_2Cl_2, dried with anhydrous Na_2SO_4. The crude product was purified by silica gel column chromatograph using dichloromethane/petroleum ether (1:1, v/v) as the eluent to obtain PDI-1. (1.05 g, 66% yield).

^{1}H NMR (400 MHz, $CDCl_3$) δ 8.26 (s, 4H), 7.05 (b, 8H), 6.72 (b, 4H), 6.53 (b, 4H) 5.09 (m, 2H), 2.28 (m, 4H), 2.05 – 1.88 (m, 4H), 1.38 (s, 36H), 1.03 – 0.95 (m, 12H). MS (MALDI-TOF): cacld for $C_{74}H_{78}N_2O_4$, 1058.60; found, 1058.36.

4) Synthesis of PDI-2

The synthesis method is the same as PDI-1.

^{1}H NMR (400 MHz, $CDCl_3$) δ 8.31 (s, 4H), 7.21 (d, *J* = 7.9 Hz, 4H), 7.05-6.97 (m, 4H), 6.80-6.37 (m, 8H), 5.08 (m, 2H), 2.37 – 2.22 (m, 4H), 1.90 (b, 4H), 1.41 – 1.23 (m, 12H), 1.00-0.87 (m, 36H). MS (MALDI-TOF): cacld for $C_{74}H_{78}N_2O_4$, 1058.60; found, 1058.53.

5) Optimized synthesis of MAS-5

PDI-1 (200 mg, 0.19 mmol), KOH (550.8 mg, 9.82 mmol) and tert-butanol (30 mL) were added into a 100 mL round bottom flask, condensed to reflux 6 hours. Then 10 mL of concentrated hydrochloric acid and 10 mL of water were added, then stirring

for another 30 min. The mixture gradually changed from a transparent orange solution to a dark green solution, and solid particles appeared at the bottom of the flask. The product was filtered and dried. The crude product was purified by silica gel column chromatograph using dichloromethane/petroleum ether (2:1, v/v) as the eluent to obtain compound 5. (156 mg, 89.7% yield)

Compound 5 (150 mg, 0.163 mmol) was suspended in 10 mL glacial acetic acid. Then o-phenylenediamine (38.8 mg, 0.358 mmol) was added and the mixture was heated to reflux for 6 hours. After cooling to room temperature, the precipitate was filtered off and rinsed with plenty of water. The crude product was purified by silica gel column chromatograph using dichloromethane/petroleum ether (2:1, v/v) as the eluent to obtain compound MAS-5. (147 mg, 84.7% yield)

^{1}H NMR (400 MHz, $CDCl_3$) δ 8.63-8.59 (m, 4H), 8.53 (2H), 7.95-7.92 (m, 2H), 7.53-7.51 (m, 4H), 7.09 (b, 8H), 6.72 (b, 8H), 1.39 (m, 36H). MS (MALDI-TOF): cacld for M-, 1064.50; found, 1065.42.

6) Optimized synthesis of MAS-6.

The synthesis method is the same as MAS-5

^{1}H NMR (400 MHz, $CDCl_3$) δ 8.68 – 8.61 (m, 4H), 8.56 (2H), 7.96 – 7.94 (m, 2H), 7.53 – 7.55 (m, 4H), 7.27 (m, 3H), 7.12 – 6.46 (m, 13H), 1.35 (s, 12H), 1.02 (s, 24H). MS (MALDI-TOF): cacld for M-, 1064.50; found, 1065.30.

7) Optimized synthesis of MAS-7.

The synthesis method is the same as MAS-5

^{1}H NMR (400 MHz, $cdcl_3$) δ 8.58 (s, 2H), 8.52 (2H), 8.48 – 8.41 (m, 2H), 7.72-

7.68 (m, 2H), 7.09 (b, 8H), 6.68 (b, 8H), 1.38 (m, 36H). MS (MALDI-TOF): cacld for M-, 1136.47; found, 1137.26.

Measurements and Instruments:

1) General Information.

^{1}H NMR spectrum was obtained on a Bruker AVANCE III 400 (400 MHz nuclear magnetic resonance (NMR) spectroscope).

EPR spectra were obtained on the Bruker A300 electron paramagnetic resonance (EPR) spectrometer.

2) Optical characterizations.

UV-vis absorption spectra were recorded on a PerkinElmer Lambda 35 spectrophotometer. Molar absorption coefficient test: according to Beer-Lambert Law: $A=\varepsilon bc$, where A is the absorbance; ε is the molar absorption coefficient; b is the thickness of the liquid tank with the unit of cm; c is the solution concentration with the unit of mol L^{-1}.

3) Electrochemical characterizations.

Cyclic voltammetry (CV) was done on a CHI604E electrochemical workstation with glassy carbon electrode, platinum electrode, and standard $Ag/AgNO_3$ as working electrode, counter electrode, and reference electrode, respectively, in a 0.1 mol/L tetrabutylammonium-hexafluorophosphate (Bu_4NPF_6) acetonitrile solution. Ferrocenium/Ferrocene (Fc/Fc+) couple was chossen as the inner reference. The equation of $E_{LUMO/HOMO} = -e\ (E_{red/ox} - E_{Fc/Fc+} + 4.8)$ (eV) was used to calculate the LUMO and HOMO levels.

For the measurement of polymer HOMO energy level, the polymer solution is uniformly spin-coated on ITO glass and used as the working electrode. Then the energy

level of the material is measured by the general method, and its LUMO energy level is calculated by its optical band gap.

4) DFT calculations

Gas-phase B3LYP/6-31G (d, p) ground-state equilibrium geometry optimizations were considered within Gaussian 09. To reduce the computational cost, all alkyl substituents were truncated to methyl groups. Molecular dihedral angles were systematically altered to ensure that the optimized geometric lower energy minimum was not missed structure possessed no imaginary frequencies. The resulting structure was characterized through frequency calculations (at the same level of theory).

5) Solar cell fabrication and testing.

The inverted devices were fabricated with the structure of ITO/ZnO/active layer/MoO_3/Ag. The prepatterned ITO-glass substrates were sequentially cleaned in ultrasonic bath with detergent, de-ionized water, acetone and isopropanol. The oven-dried substrates were then treated by an oxygen plasma for 5 min. The ZnO precursor solution (110 mg/mL) was prepared by dissolving 0.22 g $ZnAc_2 \cdot 2H_2O$ in 2 mL 2-methoxyethanol and 0.056 mL ethanol amine and then stirred at 50 °C for at least 10 h before use. The solution was filtered with polyether sulfone (PES) filters. The ZnO precursor solution was spin-coat onto ITO substrate with spinning rate of 5000 rpm for 30 s and then annealed in ambient circumstance upon 200 °C for 60 min to form a compact ZnO layer, the film thickness was ~32 nm. The mixture of donor and acceptor (D/A ratio, 1:1, weight ratio) in chlorobenzene (CB) (concentration of 13 mg mL^{-1}) was stirred overnight at 80 °C to ensure adequate dissolution, then 0.5% chloronaphthalene

(CN) was added and mixed thoroughly, and the solution was rotated (1000 rpm, 60 s) onto the ZnO layer. A MoO_3 (10 nm) layer and an Ag layer (100 nm) electrode were sequentially deposited by thermal evaporation using a shadow mask under a vacuum of <4.0×10^{-4} pa. The effective device area, defined by the overlap region of ITO and Ag electrodes, was 0.043 cm^2.

6) Mobility measurements.

The charge carrier mobilities of the blend films were measured using the space-charge-limited current (SCLC) method. The configurations of hole-only and electron-only devices are ITO/PEDOT:PSS/Active layers/M_OO_3/Ag and ITO/ZnO/ Active layers/PDINN/Al, respectively. The charge carrier mobility was determined by fitting the dark current to the model of a single carrier SCLC according to the equation:

$$u = \frac{8}{9} \cdot \left(\frac{\sqrt{J}}{V}\right)^2 \cdot \frac{d^3}{\varepsilon_0 \varepsilon_r}$$

where μ is the charge carrier mobility, *J* is the current density, d is the film thickness of the active layer, ε_r is the relative dielectric constant of the transport medium, and ε_0 is the permittivity of free space. $V = V_{appl} - V_{bi} - V_s$, where V_{appl} is the applied voltage, V_{bi} is the offset voltage. V_s is the voltage drop due to contact resistance and series resistance across the electrodes. The carrier mobility can be calculated from the slope of the $J^{1/2} \sim V$ curves.

7) AFM analysis.

The morphologies of active layers were investigated by Park Systems NX-10 high-resolution scanning probe microscope. The specimen for AFM measurements was prepared using the same procedures for fabricating devices.

8) TEM analysis.

Transmission electron microscopy (TEM) measurement was performed by using a HITACHI H-7650 electron microscope with an acceleration voltage of 100 kV.

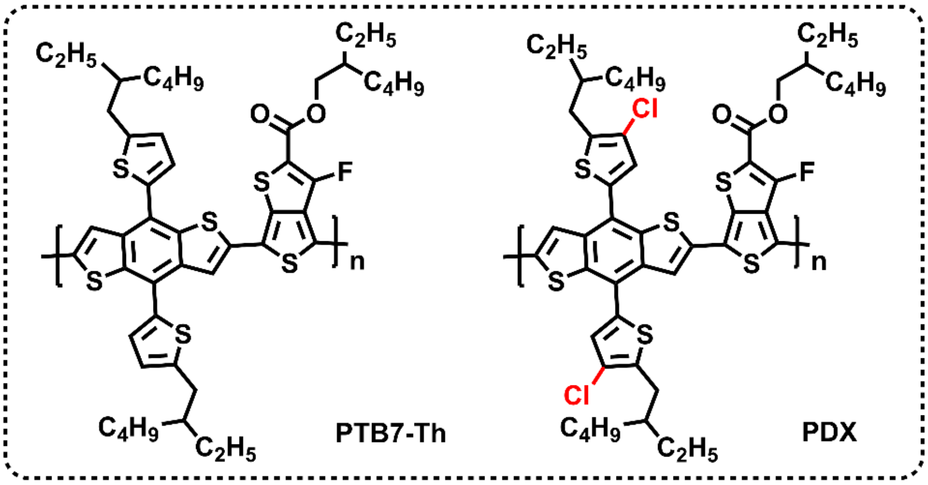

Figure S1. Molecular structure of the donor materials PTB7-Th and its chlorinated derivative PDX

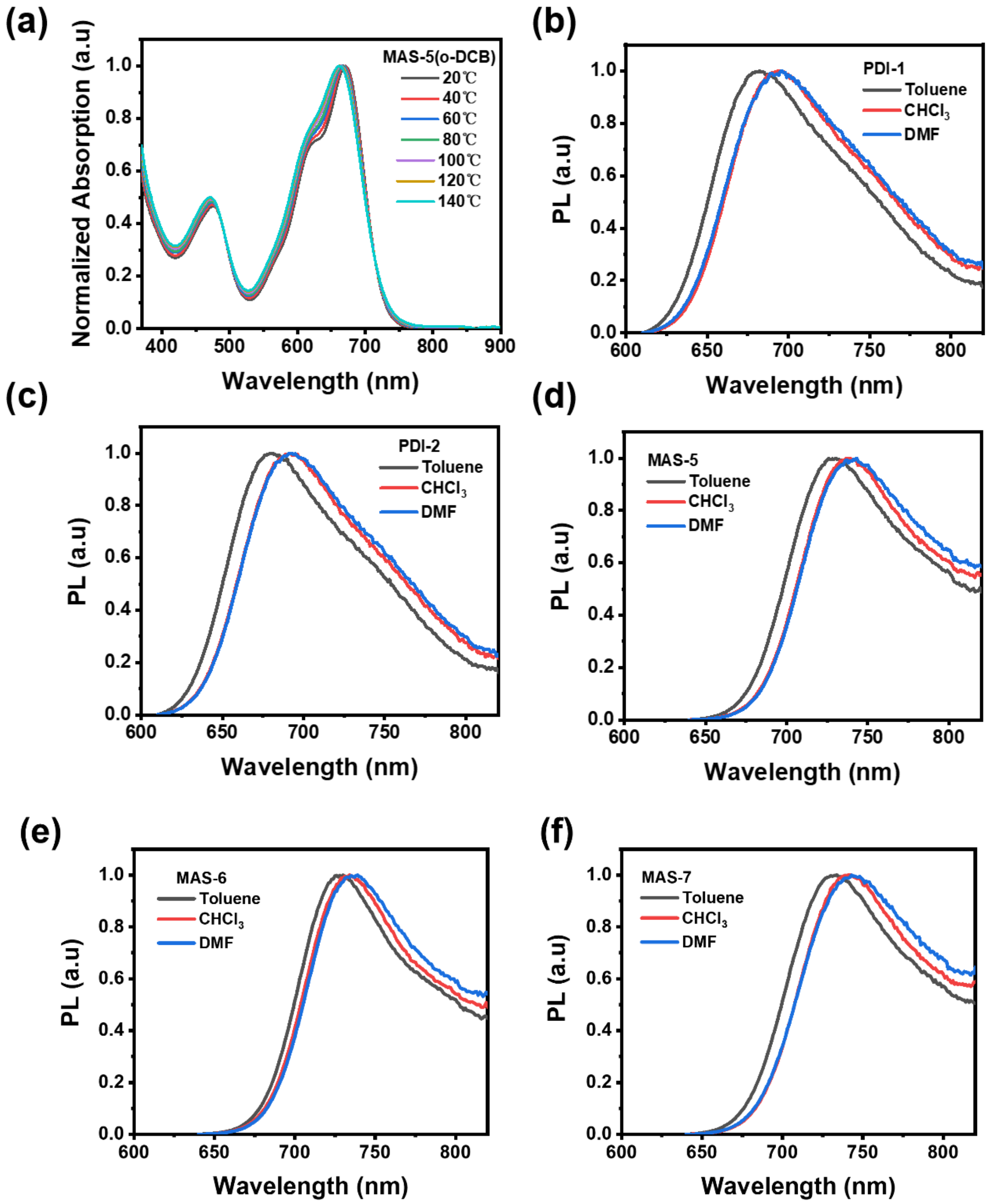

Figure S2. (a) Normalized temperature-dependent UV-vis spectra of the MAS-5. The Steady photoluminescence spectra in toluene, chloroform (CF) and N,N-Dimethylformamide (DMF) solution of (b) PDI-1, (c) PDI-2, (d MAS-5, (e) MAS-6 and (f) MAS-7.

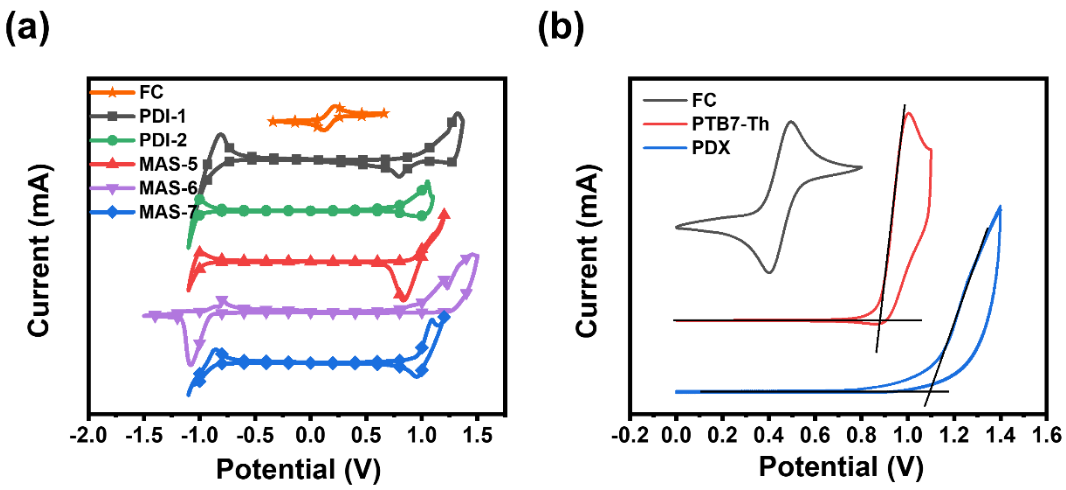

Figure S3. (a) Cyclic voltammogram (CV) curves of PDI-1, PDI-2, MAS-5, MAS-6 and MAS-7. (b) Cyclic voltammetry measurement of thin films of PTB7-Th and PDX.

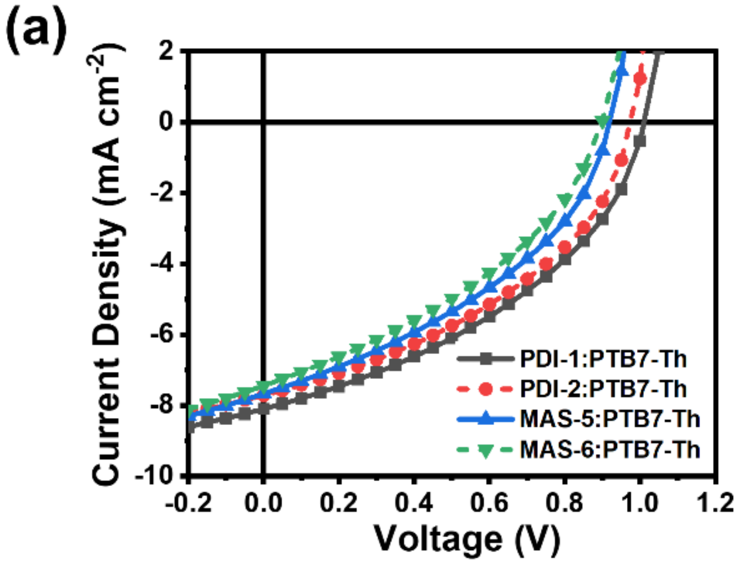

Figure S4. (a) The J-V curves spectra of the OSCs based on PTB7-Th.

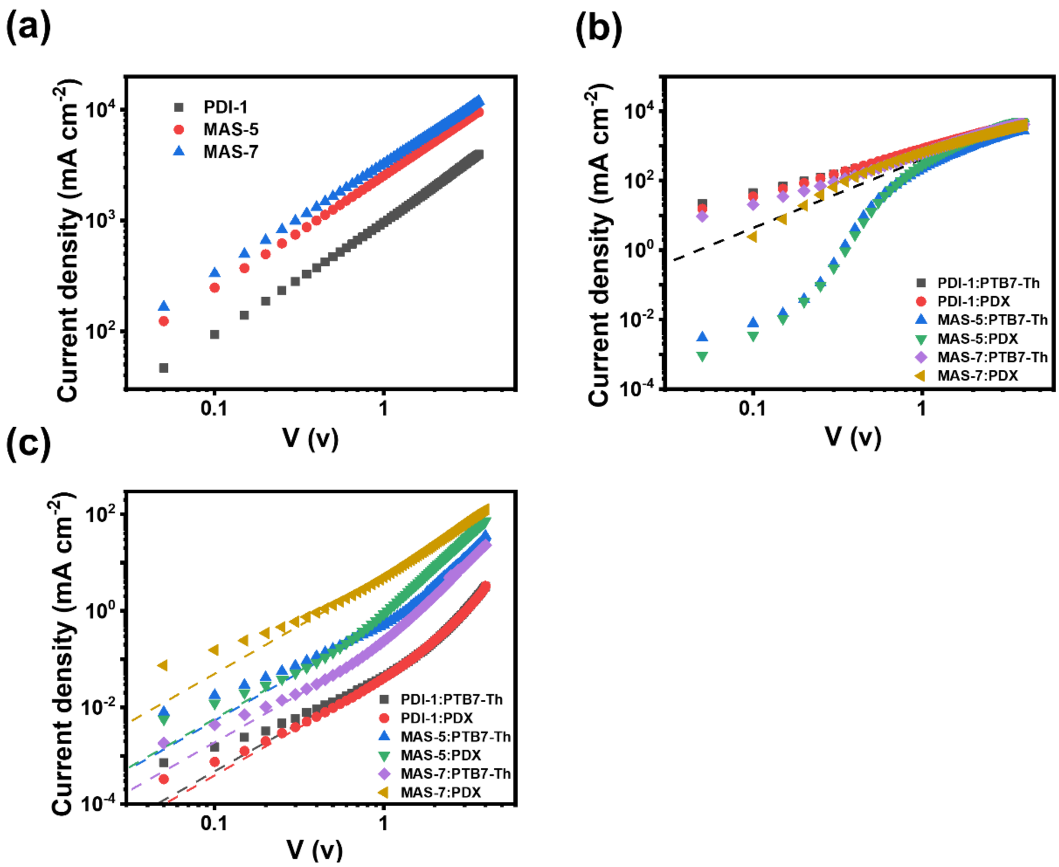

Figure S5. (a) electron mobilities of the electron-only devices with pure acceptor films. (b) hole mobilities for the hole-only devices; (c) electron mobilities for the electron-only devices.

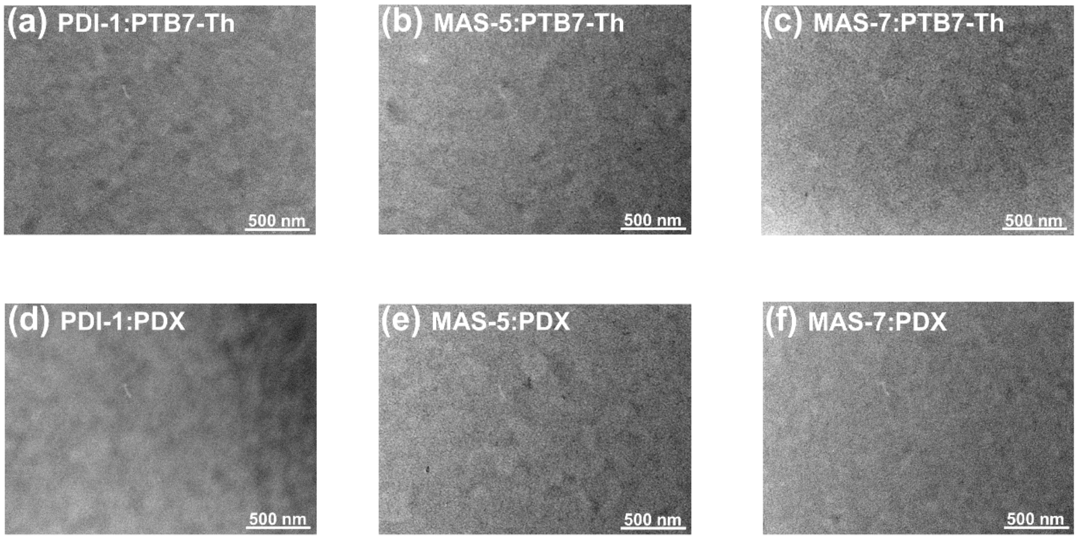

Fig. S6. TEM images for films of (a) PDI-1:PTB7-Th, (b) MAS-5:PTB7-Th, (c) MAS-7:PTB7-Th, (d) PDI-1:PDX, (e) MAS-5:PDX, and (f) MAS-7:PDX blend.

Table S1. The HOMO level of the PTB7-Th or PDX measured by use of cyclic voltammetry (Fig. S2d).

Sample structure	HOMO[a] (eV)	Optical band gap[b] (eV)	LUMO[c] (eV)
**PTB7-Th**	-5.23	1.62	-3.61
**PDX**	-5.42	1.70	-3.72

a. Determined by CV.

b. Optical band gap is taken from UV-vis absorption onset (Fig. 2b).

c. E_{LUMO} is determined by the relation: $E_{LUMO} = E_{HOMO} + E_g^{opt}$

Table S2 The calculated HOMO/LUMO energy levels for PDI-1, PDI-2, MAS-5, MAS-6 and MAS-7

	PDI-1	PDI-2	MAS-5	MAS-6	MAS-7
**HOMO**	-5.39	-5.43	-5.24	-5.29	-5.41
**LUMO**	-3.15	-3.19	-3.20	-3.24	-3.39

Table S3 Key parameters of OSCs based on PTB7-Th:PDI-2/MAS-6.

Donor	Acceptor	J_{sc} ($mAcm^{-2}$)	V_{oc} (V)	FF	PCE
**PTB7-Th**	**PDI-2**	**7.71**	**0.97**	**41.58**	**3.12**
	**MAS-6**	**7.44**	**0.90**	**38.09**	**2.55**

Table S4 The charge mobility of PTB7-Th/PDX:PDI-1/MAS-5/ MAS-7 devices.

Blend films	μ_h (cm^2 V^{-1} s^{-1})	μ_e (cm^2 V^{-1} s^{-1})
**PDI-1**		4.06×10^{-5}
**PDI-1:PTB7-Th**	7.51×10^{-5}	3.39×10^{-7}
**PDI-1:PDX**	4.39×10^{-5}	4.63×10^{-7}
**MAS-5**		1.03×10^{-4}
**MAS-5:PTB7-Th**	12.8×10^{-5}	1.40×10^{-6}
**MAS-5:PDX**	9.41×10^{-5}	4.13×10^{-6}
**MAS-7**		1.21×10^{-4}
**MAS-7:PTB7-Th**	8.44×10^{-5}	1.46×10^{-6}
**MAS-7:PDX**	6.78×10^{-5}	4.67×10^{-6}
